# Activation of VIP signaling enhances immunosuppressive effect of MDSCs on CMV-induced adaptive immunity

**DOI:** 10.18632/oncotarget.20704

**Published:** 2017-09-07

**Authors:** Parvin Forghani, Christopher T. Petersen, Edmund K. Waller

**Affiliations:** ^1^ Department of Hematology and Medical Oncology, Winship Cancer Institute, Emory University, Atlanta, Georgia, USA

**Keywords:** CMV, myeloid-derived suppressor cells (MDSC), VIP, mice, Immunology and Microbiology Section, Immune response, Immunity

## Abstract

Vasoactive intestinal peptide (VIP) is recognized as a potent anti-inflammatory factor which affects both the innate and adaptive arms of the immune system. These effects include, but are not limited to, inhibition of T cell proliferation and disruption of immune homeostasis. Myeloid-derived suppressor cells (MDSC) are an immune regulatory cell type that has been described in settings of cancer and infectious disease._Here we demonstrate a reduced circulating monocytic MDSCs in the VIP ^-/-^ *vs.* wild type MCMV. VIP-/- MDSCs secretes less NO upon stimulation with LPS and interferon that relatively lose the ability to suppress T cells activation *in vitro* compared to wild type MDSCs._Considering the importance of VIP in immunomodulation, the possible effect of VIP in the suppressive function of MDSC populations following CMV infection remains unknown. We describe the possible role of VIP in the regulation of anti-CMV activity of T cells through the activation of MDSCs.

## INTRODUCTION

MDSC are identified as monocytic and granulocytic- CD11b^+^ GR-1^+^ populations of immature myeloid cells that are expanded in malignant states, bacterial infections, and fungal infections [1-3]. They play a vital role in disease modulation of cancer and chronic inflammatory illnesses through suppression of CD4^+^ and/or CD8^+^ T cell function [4-6].

Vasoactive intestinal peptide (VIP) is an important immunosuppressive neuropeptide that can influence a broad range of immune cells [7-9]. VIP has a key role as a potent anti-inflammatory factor as identified by its ability to inhibit T cell proliferation [10]. Additionally, VIP inhibits the production of inflammatory cytokines and chemokines from macrophages, microglia and dendritic cells through the VIP receptors VPAC1 and VPAC2 [10]. These receptors are widely distributed throughout the body, including cells of the immune system and VPAC1 up regulated by inflammation [9] . VPAC1 expression has been identified on murine lymophocytes and rat peritoneal macrophages [11, 12]. It is evidenced that inflammation induces the expansion of MDSC populations in tumor-bearing mice and infected animals [13]. In these settings, MDSCs contribute to tumor-associated antigen-specific T cell dysfunction and tolerance [6, 14].

In the case of infectious diseases, and more specifically CMV infection, little is known about the effect of VIP on CD11b^+^ GR-1^+^ cells. It has also been reported that VIP interferes with control of innate and adaptive immune responses [15]. Recently, the effect of VIP on T regulatory cells was described [16] . However, there has been no description of the possible effects of VIP on MDSCs number and function. To understand the function of MDSCs involved in the regulation of inflammation associated with CMV infection, we used CMV infected VIP^−/−^ *vs*. wild type mice. We document herein that signaling through the VIP pathway enhanced the suppressive activities of MDSC, limiting adaptive immune responses to CMV.

## RESULTS

### Reduced circulating monocytic MDSCs subunit upon CMV infection in VIP^−/−^ mice *vs.* wild type

**Figure 1:**
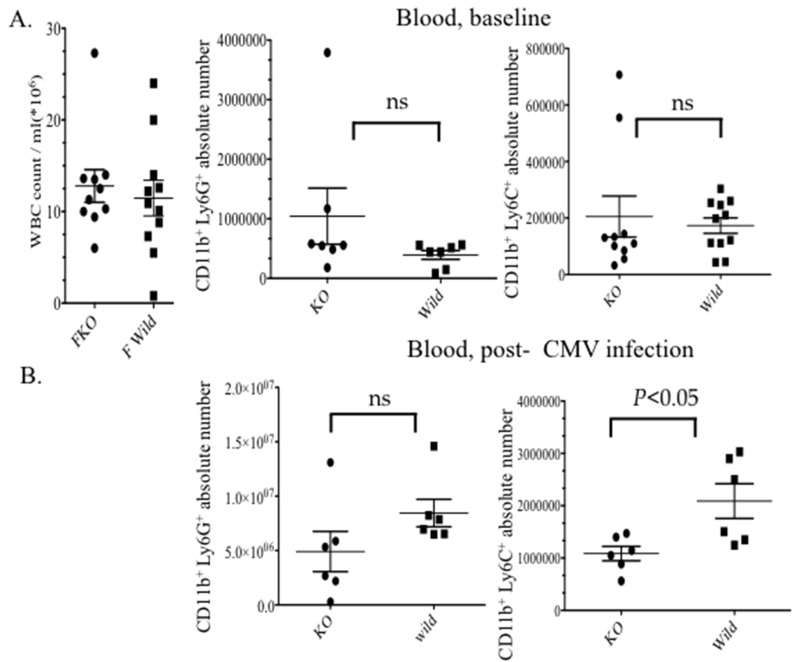

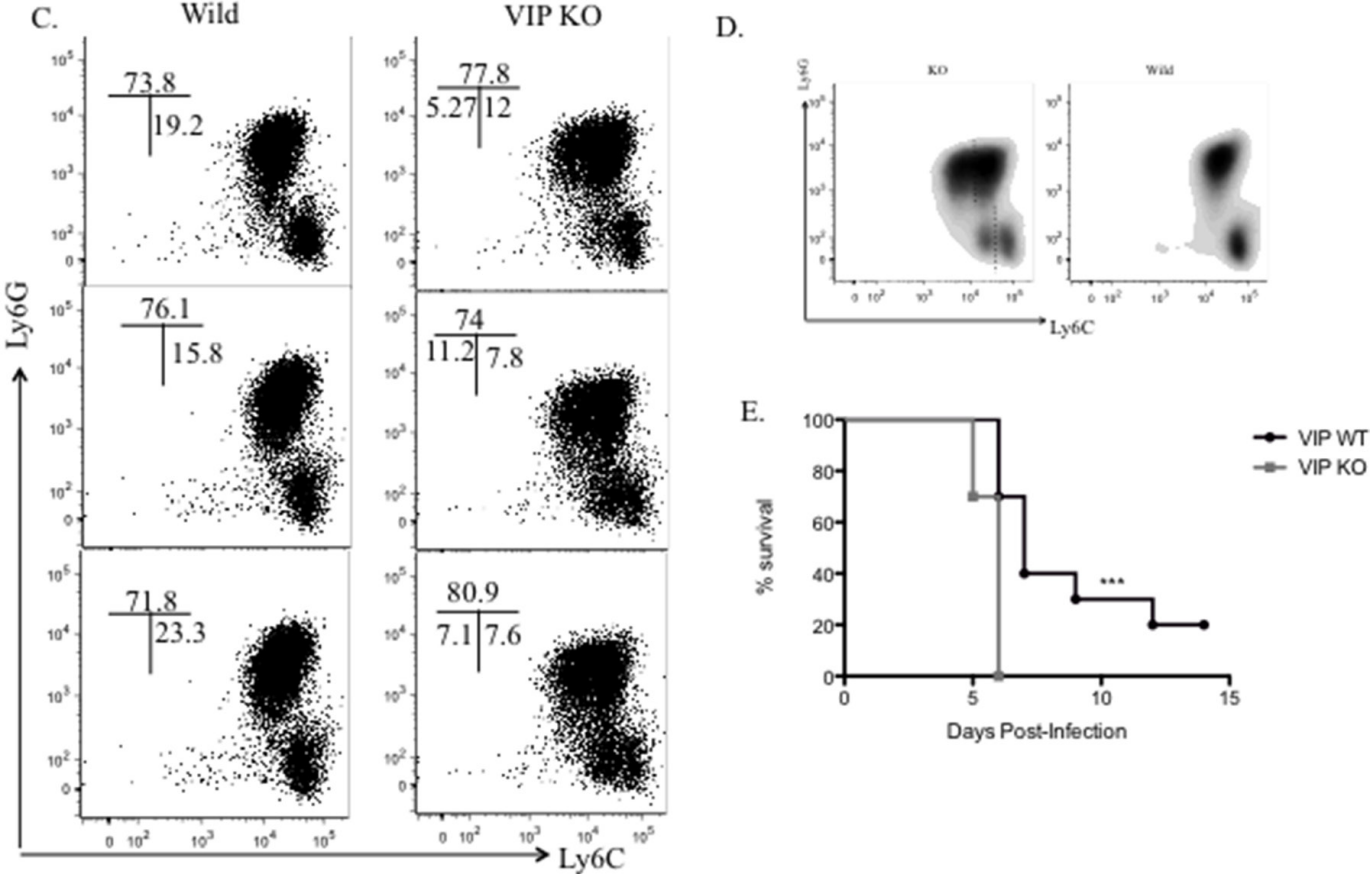
Decreased recruitment of monocytic MDSCs in the blood of VIP^−/−^ mice 4 days post- CMV infection Comparison of IMCS and MDSCs subsets in blood in both uninfcted **A.** and infected **B.** VIP^−/−^and wild type mice. **C.**, **D.** Characterization of MDSC subsets by flow cytometry. FACS dot plots showing MDSC population in the peripheral blood of VIP^−/−^and wild type mice post CMV infection. (*n* = 4 mice/ time point, three replicate experiments). **E.** Survival of female VIP^−/−^ and wild type mice in response to high dose MCMV infection. (*n* = 10 mice per group).

To investigate the effect of VIP signaling on MDSCs during MCMV infection, we checked the frequency and absolute number of CD11b^+^Gr1^+^ cells in the blood of VIP knockout *vs*. wild type mice. Blood was collected from both groups 4 days post CMV infection. There were no significant differences in the absolute number and frequency of immature myeloid cells (IMC) or total white blood cell count (WBC) in the blood when comparing VIP^/−^ mice to wild type at baseline (Figure 1A). Although decreased absolute numbers of CD11b^+^Gr1^+^ MDSCs in the blood of VIP^−/−^ mice were observed, the decrease was not statistically significant. However, a statistically significant difference was observed in the frequency of the monocytic (Lymphocyte- antigen 6) Ly6C MDSC subpopulation (Figure 1B). Analyzing the phenotypes of sub populations of MDSC showed a population of Ly6C^lo/med^ cells in VIP^−/−^ mice that was not observed in the wild type counterpart. Both VIP^−/−^ and wild type mice had an increase in Ly6G^+/hi^ granulocytic MDSCs following MCMV infection (Figure 1C-1D). In order to determine whether the decrease in Ly6C^+^ MDSCs in VIP^−/−^ mice was due to differences in bone marrow production, we characterized the MDSC populations in the bone marrow 4 days after infection by flow cytometry. Our results didn’t show any significant difference in the frequencies of MDSC populations in the marrow (Supplementary Figure 1). Interestingly, female VIP^−/−^ mice were significantly more susceptible to high dose MCMV infection (Figure 1E). In accordance with these results, our findings confirmed that lower absolute numbers of monocytic MDSCs can lead to lower survival in female VIP^−/−^ MCMV infected mice.

### Absence of VIP signaling lead to partial inhibition of MDSC-mediated T cell suppression

**Figure 2:**
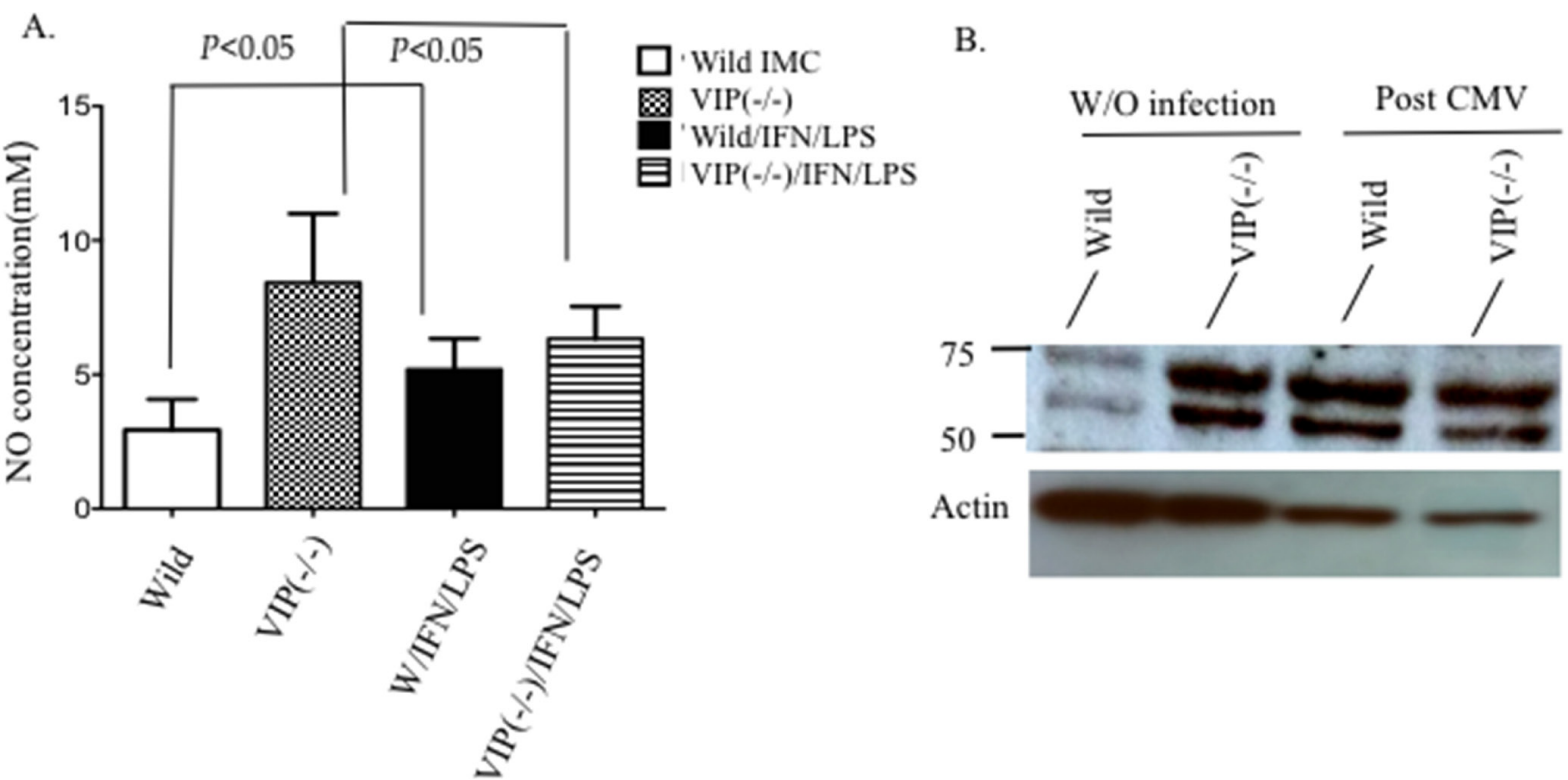

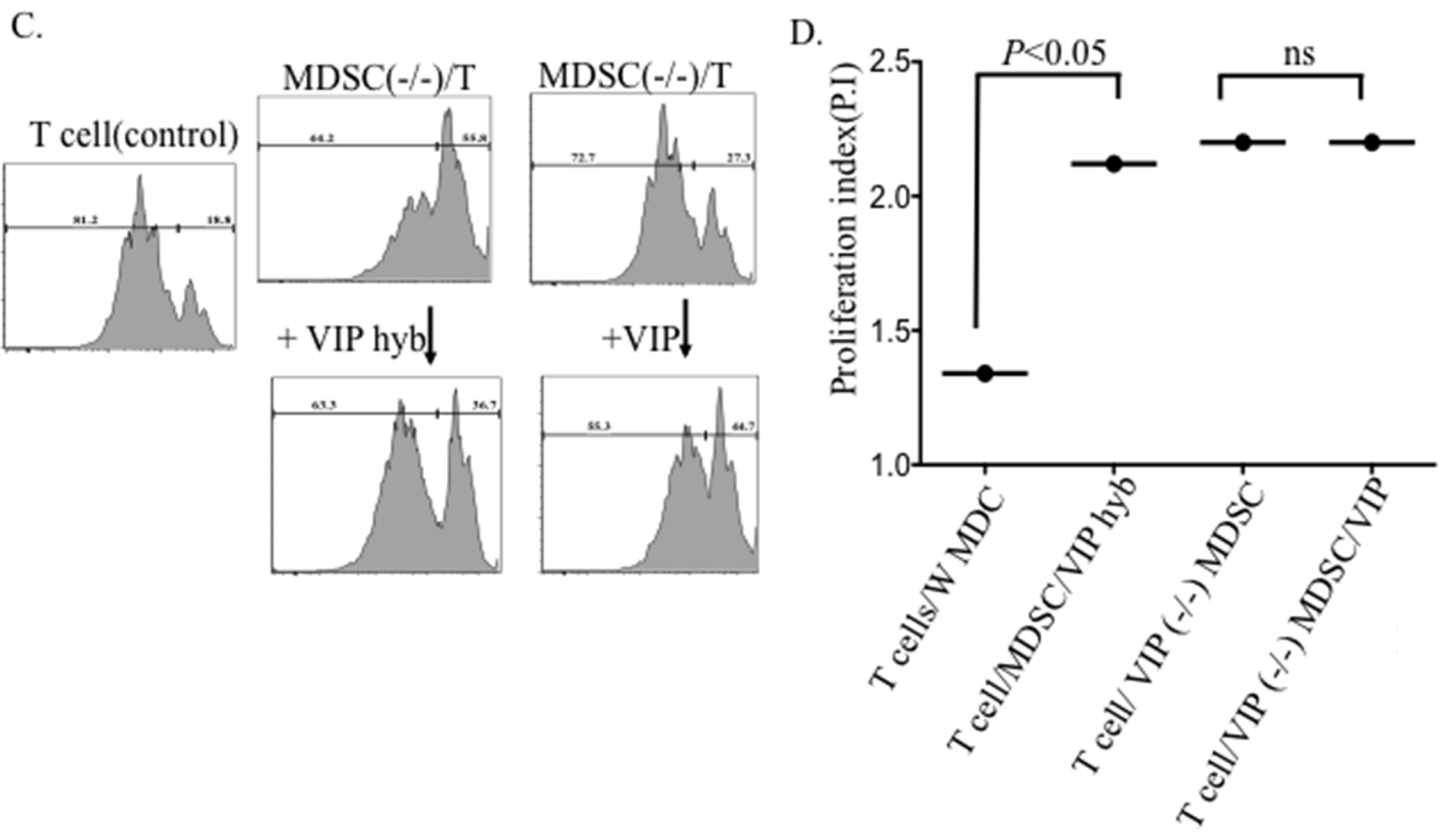
Production of VIP by MDSCs contributes to the immunosuppressive function To confirm a reduction in the suppressive activity of VIP^−/−^ MDSCs, BM-derived MDSCs were tested for their capacity to secrete nitric oxide and to inhibit T cell proliferation. **A.** Quantitation of nitric oxide production in wild type and VIP ^−/−^ IMCs following IFN-γ and LPS stimulation. **B.** CFSE proliferation assay of wild type T cells cultured in the presence or absence of VIP^−/−^ or wild type MDSC with or without VIP and with VIP antagonist (VIPhyb). Undivided cells *vs*. divided cells, from two experiments **C.** Comparison of the proliferation indexes in each culture system. **D.** Western blot showing expression of VPAC1 protein in wild-type and VIP^−/−^ MDSCs. *Two-way Anova*. (*p < 0.05*).

To determine if VIP plays an important role in the immunosuppressive activity of MDSCs, we tested the effect of VIP signaling on IMCs in wild type and VIP ^−/−^ mice. First we tested the capacity of sorted BM-derived IMCs harvested from both WT and VIP ^−/−^ mice to release NO upon stimulation with IFN-γ and LPS. BM-derived IMCs from both VIP^−/−^ and wild type were cultured for 19 hours in the presence or absence of IFN- γ and LPS. VIP^−/−^ IMCs, released significantly less NO than WT IMCs upon stimulation with IFN-γ and LPS suggesting a possible role of VIP in the immunosuppressive activity of MDSCs (Figure 2A). It has been established that VPAC1 is constitutively expressed on lymphocytes, dendritic cells, and macrophages [12, 17]. To examine the pattern of VPAC1 expression on IMC subpopulations, FACS-sorted BM cells were obtained from wild type and VIP^−/−^ mice. As shown in Figure 2B, VPAC1 expression is upregulated on wild type MDSCs following MCMV infection while higher levels of VPAC1 are expressed in VIP^−/−^ mice at baseline. Our results indicate that that VIP is negative regulator of VPAC1 expression on immature myeloid cells in the absence of viral infection.

To directly test the functional role of VIP in MDSC-mediated T-cell suppression, sorted BM-derived CD11b^+^GR-1^+^ IMCs from wild type and VIP^−/−^ were co-cultured with CFSE-labeled wild type T cells as described in the methods. Signaling through VPAC1 on wild type T/MDSC co-cultures was blocked using the peptide antagonist VIPhyb. T cells in plates containing bound VIP or peptide antagonist of VIP (VIPhyb) cultured with VIP^−/−^ MDSC proliferated more than T cells cultured with wild type MDSCs. Addition of exogenous VIP to cultures of T cells with VIP^−/−^ MDSCs did not inhibit T cell proliferation in culture with wild type MDSCs. In contrast, addition of VIPhyb to wild type BM-derived MDSCs co-cultures gave results that mimicked the augmented T cell proliferation seen in cultures containing VIP^−/−^ MDSCs (Figure 2C-2D). Our results demonstrate VIP^−/−^ MDSCs secrete less NO upon stimulation with LPS and interferon and VIP ^−/−^ MDSCs have an impaired ability to suppress T cells *in vitro* compared to wild type MDSCs. Additionally, splenocytes from both VIP^−/−^ and wild type mice were cultured with IFN-γ and LPS and stained for intracellular pro-inflammatory cytokines that involved in NO production (Supplementary Figure 2). Our results confirmed significant decrease in TNF cytokine production in VIP^−/−^*vs*. wild type mice.

## DISCUSSION

The present study examined the possible roles of VIP in the immune-regulatory role of myeloid-derived suppressor cells during CMV infection. VIP has been identified as a potent anti-inflammatory factor, which acts by regulating the production of both anti- and pro-inflammatory mediators [12, 17] . Importantly, several studies have identified VIP as a potent suppressor of T cell activation and proliferation [18, 19].

It has previously been shown that MDSCs in the tumor microenvironment are capable of inhibiting activation and proliferation of T cell cells [20, 21], however, the role of MDSCs and VIP during viral infection is has not been previously characterized. Our data confirms that CMV infection induces significantly fewer monocytic MDSCs in the absence of VIP. The significance of this finding is highlighted by a previous study which determined that the mobilization of inflammatory monocytic cells is considered a hallmark response to CMV infection that serves to limit pathological consequences of viral infection [22]. Further, our data confirms that lower frequencies of MDSCs in the blood of VIP^−/−^ mice were not the result of decreased bone marrow generation of these cells.

As our results show, VIP^−/−^ MDSCs produce significantly less NO compared to wild type-derived MDSCs following stimulation with IFN-γ and LPS. The significant difference in NO production supports the role of VIP in NO production and the expression of functional VIP receptors on MDSCs. Although VIP^−/−^ -derived MDSCs showed less potential to suppress T cell proliferation *in vitro*, addition of exogenous VIP to VIP^−/−^ MDSC/T co-cultures couldn’t inhibit the proliferation of T cells to levels seen with wild-type MDSCs.

Expression of VPAC1 on IMCs/MDSCs provides a new potential mechanism of MDSC-mediated anti-inflammatory effects following CMV infection. It has been previously reported that VIP is a negative regulator of VPAC1 expression [23]. In accordance with these studies, the expression of VPAC1 on IMCs was lower in the presence of VIP. However, following CMV infection, VPAC1 expression on wild type MDSCs was similar to that on VIP^−/−^ derived MDSCs indicating an upregulation of the receptor in response to viral infection. Considering the established role of endogenously regulated expression of VIP as a mediator for VPAC1 internalization [12], differences in the secretion of VIP in response to CMV infection could account for this observation.

In summary, our work describes previously unknown mechanism by which lack of VIP signaling limits the migration of monocytic MDSC subsets in blood following CMV infection. The role of VIP as a modulator of the number and function of MDSCs might broaden the potential clinical application of VIP inhibitors in cancer immunotherapy. Our data shows that a lack of VIP signaling results in partial inhibition of immune suppression and supports a possible role for VIP-induced modulation of MDSCs. Based on the results presented above, we propose that the effects of VIP on immune responses may result from indirect effects on IMCs/ MDSCs.

## MATERIALS AND METHODS

### Mice and CMV infection model

VIP knockout^−/−^ mice were obtained from Walshock [24]. The genotype of wild type heterologous and homologues VIP knock out mice were determined by PCR of genomic DNA [24] and bred as heterozygotes at Emory University. Sex and age-matched wild type litter mates were used as controls. Infections of mice were performed by intraperitoneal injection of 10^6^ pfu of Smith strain murine cytomegalovirus (MCMV). All experiments were approved by the Emory University Institutional Animal Care and Use Committee.

### Cell sorting

Purification of MDSCs (CD11b^+^, Gr-1^+^) was performed by harvesting bone marrow from the femurs of mice as described previously [25]. Whole bone marrow was then stained, and purified MDSC populations were recovered using a BD FACS Aria or a MACS column using anti-PE microbeads (Miltenyi Biotec, Bergisch Gladbach, Germany).

### Flow cytometry

The phenotype of myeloid cells was determined using multi-color flow cytometry as previously reported [25-27]. Dead cells were excluded in analysis with SYTOX blue (Thermo fisher). All staining was performed in the presence of 10 μg/ml Fc-Block (BD biosciences). Fluorochrome-labeled isotype controls were used as indicated. For the enumeration of total cell numbers in blood, an equal volume of counting beads was added to each sample as described previously [27]. Cell sorting was performed using a BD FACS Aria (BD Bioscience, San Joes, CA). List mode files were analyzed using FlowJo software (TreeStar, Inc, Ashland, OR).

### T cell suppression assay

MACS-purified T cells were labelled with 0.1μM carboxyfluorescein-succinimidyl-ester (CFSE) and stimulated with mouse T-activator CD3/CD28 Dynabeads (Life Technologies, Darmstadt, Germany). Different subpopulations of sorted myeloid cells were co-cultured with the T cells at the indicated ratios in the presence of 30 U/mL IL-2. Proliferation of T cells was analyzed after 72h co-culture by quantitating CFSE dilution with flow cytometry. 200 microliters of VIP and VIPhyb (150 microgram) was used to cover 24 wells plates for overnight at 4 degrees.

### *in vitro* stimulation

Sorted BM cell were obtained from uninfected wild type and VIP^−/−^, were cultured in 24 well plates (3 × 10^5^ cells per well) and stimulated with IFN gamma and LPS (1mg/ml,100 microgram/ml) in RPMI for 16 hrs. Supernatant was collected to measure NO as described [25].

### Western blotting

Protein lysates were prepared from sorted MDSC populations using cell lysis buffer containing 1 mM PMSF (Cell Signaling Technologies, Danver, MA). For immunoblot analyses, 10- 30 μg samples of protein were loaded onto 10% SDS-PAGE gel separated by electrophoresis and transferred to a nitrocellulose membrane (BioRad, Hercules, CA). Membranes were blocked with 5% milk for 1 hour at room temperature and probed with 1:2000 dilutions of polyclonal anti-VPAC1 antibodies overnight at 4°C (Santa Cruz Biotechnologies, Dallas, TX). Blots were then incubated with a secondary peroxidase-conjugated antibody at a 1:3000 dilution. Band visualization was performed using a chemiluminescent HRP substrate (Millipore, Billerica, CA) followed by exposure to X-ray film. Bands between 37-60 KD were considered as positive for VPAC1 expression. Membranes were then stripped and re-probed with an anti-actin antibody (Cell Signaling Technologies, Danvers, MA) at a 1:3000 dilution to serve as a control for protein loading and transfer.

### Evaluation of NO production

The functional status of T cells was evaluated by measuring extracellular nitric oxide production as previously described [25]. Briefly, equal volumes of culture supernatants (100 μl) were mixed with Greiss reagent. After 15-30 min of incubation at room temperature, the absorbance at 550 nm was measured using a microplate plate reader (Bio-Rad, Hercules, CA). Nitrite concentrations were determined by comparing the absorbance values for the test samples to a standard curve generated by a serial dilution of 0.25 mM sodium nitrite.

## SUPPLEMENTARY MATERIALS FIGURES



## Abbreviations

MDSC: Myeloid-derived suppressor cells
VIP-/-: knock out
FACS: fluorescent activated cell sorting
