# Supplementary material for: Activation of VIP signaling enhances immunosuppressive effect of MDSCs on CMV-induced adaptive immunity
Source: Oncotarget. 2017 Sep 7;8(47):81873–9. doi: 10.18632/oncotarget.20704 (PMC5669855; doi:10.18632/oncotarget.20704)
Supplement: Supplementary file 1 [file oncotarget-08-81873-s001.pdf]

# Activation of VIP signaling enhances immunosuppressive effect of MDSCs on CMV-induced adaptive immunity

## SUPPLEMENTARY MATERIALS

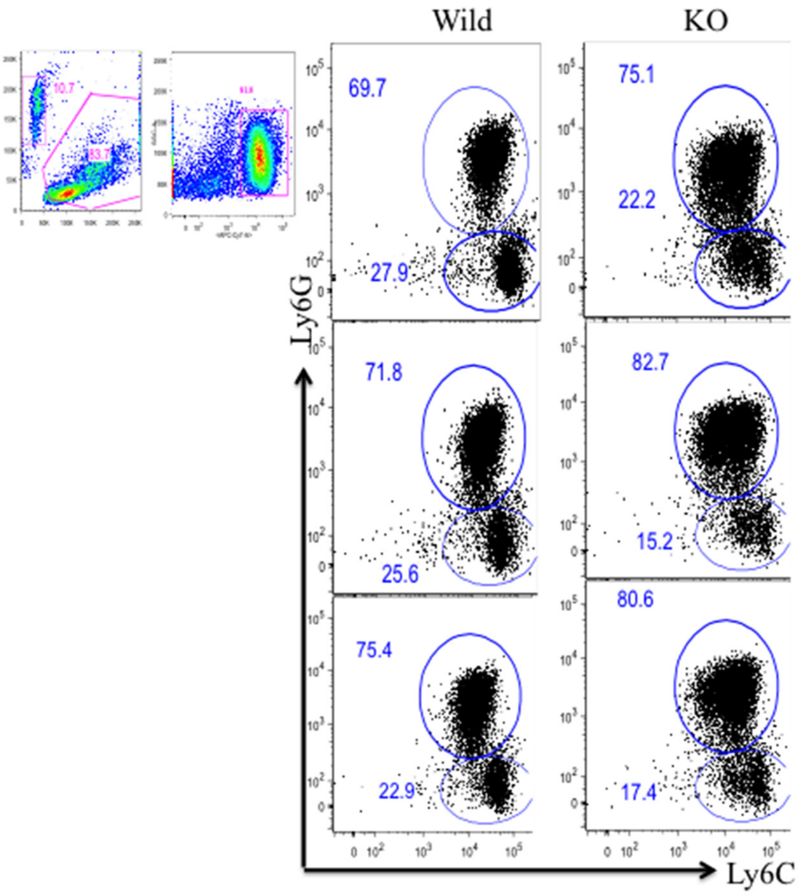


**Supplementary Figure 1: Frequencies of IMC and MDSC subpopulations in single cell suspensions from wild type and VIP$^{-/-}$ bone marrow.**

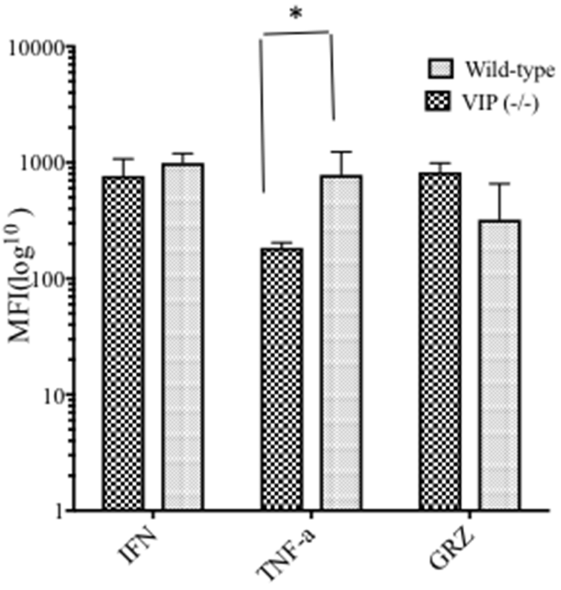


**Supplementary Figure 2: Pro-inflammatory cytokine production in VIP wild type and VIP $^{-/-}$ splenocytes following stimulation with IFN-γ and LPS .**
